# Supplementary material for: Effects of Protein Supplementation Combined with Exercise Training on Muscle Mass and Function in Older Adults with Lower-Extremity Osteoarthritis: A Systematic Review and Meta-Analysis of Randomized Trials
Source: Nutrients. 2020 Aug 12;12(8):2422. doi: 10.3390/nu12082422 (PMC7468926; doi:10.3390/nu12082422)
Supplement: Supplementary file 1 [file nutrients-12-02422-s001.pdf]

Supplementary Table S1

Table S1. Database search formulas

Data base	Search terms for query
**Pubmed**	
#1	(elder adults) or older adults
#2	osteoarthritis
#3	arthroplasty or (total joint replacement)
#4	#1 and ((#2) or #3)
#5	((whey protein) or (amino acid) or (leucine)) supplementation
#6	((nutrition) or (nutrient)) intervention
#7	#5 or #6
#8	(exercise training) or (physical activity)
#9	(randomized controlled trial) or randomization
#10	#4 and #7 and #8 and #9
**Physiotherapy Evidence Database (PEDro)**	
	Method: clinical trial
	Abstract and Title:
#1	elderly
#2	osteoarthritis
#3	arthroplasty
#4	exercise training
#5	physical activity
#6	protein supplementation
#7	nutrition intervention
**Excerpta Medica dataBASE (EMBASE)**	
#1	osteoarthritis
#2	arthroplasty
#3	exercise training
#4	physical activity
#5	protein supplementation
#6	nutrition intervention
#7	#1 or #2
#8	#3 or #4
#9	#5 or #6
#10	#7 and #8 and #9 and (randomized controlled trial)/lim and (humans)/lim

(continued)

Table S1. (continued)

Data base	Search terms for query
**Cochrane Library Database**	
#1	elderly
#2	osteoarthritis
#3	arthroplasty
#4	exercise training
#5	physical activity
#6	protein supplementation
#7	nutrition intervention
#8	#1 and (#2 or #3)
#9	#4 or #5
#10	#6 or #7
#11	randomized controlled trial
#12	#8 and #9 and #10 and #11
**China knowledge resource integrated database**	
#1	(osteoarthritis) or (arthroplasty)
#2	(exercise training) or (physical activity)
#3	(protein supplementation) or (nutrition intervention)
#4	randomized controlled trial
#5	#1 and #2 and #3 and #4
**Google Scholar**	
#1	allintitle: elderly or older adults
#2	allintitle: osteoarthritis or arthroplasty
#3	allintitle: exercise training
#4	allintitle: physical activity
#5	allintitle: “protein supplementation” or “nutrition intervention”
#6	allintitle: randomized controlled trial

Supplementary Table S2

Table S2. Summary of protein supplementation protocols in the included studies

Study	Experimental group					Control group
	Protein sources	Intake amount (g/day or g/session)	Weekly servings	Supplement type	Intake timing	Source of supplement
Baldissarro 2016	Leucine, EAA	8 g/day (2 servings/day)	14	Oral EAAs (leucine 1.250 g/serving)	NR; the 14-day treatment was dictated by the rehabilitation center policy.	Placebo (maltodextrin)
Dreyer 2013; 2018	EAA	40 g/day (2 serving/day)	2	20 g master mix (leucine, 3.6 g/serving)	Daily at 10:00 AM and 14:00 PM; 1 hour after physical therapy	Placebo (non-EAA; alanine 100%, 20 g)
Ikeda 2018	BCAA	6 g /session	7	A 6-g tablet amino acid supplement (amino acids 3 g; leucine 21%); taken with 200 mL of water	Before the exercise	Placebo (1.2-g starch polysaccharide)
Ikeda 2019	BCAA	3.0 g /session	7	A 3.4-g tablet amino acid supplement (amino acids 3 g; leucine 40%); taken with 200 mL of water	After the exercise	Placebo (1.2-g starch polysaccharide)
Muyskens 2019	EAA	40 g/day (2 serving/day)	14	20-g EAA supplement mixed with any liquid or food between meals (leucine 18%, 3.6 g /serving)	Inpatient stay: Daily at 10:00 AM and 14:00 PM; 1 hour after physical therapy. Outpatient: One hour after exercise on the physical-therapy day and at the regular times on all other days.	Placebo (non-EAA; alanine 100%, 20 g)

AAS, amino acid supplementation; BCAA, branched chain amino acids; EAA, essential amino acids.

Supplementary Table S3

Table S3. Summary of exercise training protocols in the included studies

Study (Author, year)	Experimental group											Control group
	Training environment	Flexibility/ROM/ stretching exercises	Muscle strengthening exercise (progressive RET)				Endurance (AET)	Functional mobility exercises	Training time (min/session)	Frequency (session/w)	Intervention duration (wk)	Intervention
			Training part	Resistance set	Intensity	Training volume						
Baldissarro 2016	Supervised postoperative rehabilitation	Passive mobilization (hip, knee, and ankle); stretching (hip adductor and flexor muscles)	Lower extremity exercises	Isotonic contraction: hip extension (gluteus); knee extension (quadriceps)	Against a resistance of 1.0 kg	NR	Maintenance of cardiorespiratory capacity	Gait training (use of walking sticks); stair training.	45	2 session/d;	2	Exercise control group: the same exercise program as experimental group did.
Dreyer 2013; 2018	Supervised physical therapy	NR	Lower limbs (Physical therapy)	NR	NR	NR	NR	NR (Physical therapy)	NR	7	3	The same exercise program as experimental group did.
Ikeda 2018	Home-based exercise	None	Lower extremity (hip abduction and clamshell exercise)	Elastic bands; tonic force generation method (seated on the muscle training machine, 3-s eccentric, 3-s concentric, and 1-s isometric actions, with no rest between each repetition)	Low-intensity; slow movement	2 sets of 20 repetitions	None	None	NR	7	4	The same exercise program as experimental group did.
Ikeda 2019	Rehabilitation-ward exercise therapy	ROM exercises (20 min)	Lower extremity (hip abduction, clamshell exercise, and knee extension)	Elastic bands	NR	20 min	None	Gait and ADL training (20 min)	60	7	4	The same exercise program as experimental group did.
Muyskens 2019	Supervised physical therapy	NR	Lower limbs (Physical therapy)	NR	NR	NR	None	NR (Physical therapy)	NR	7	3	Theme-based sitting activities (watching films, reading, conversation)

AET, aerobic exercise training; RET, resistance exercise training; ROM, range of motion; ADL, activities of daily living; NR, not reported.

Supplementary Table S4

Table S4. Methodological quality of the included studies[a]

Study (author, year)	Overall[b]	Eligibility criteria[c]	1	2	3	4	5	6	7	8	9	10
Baldissarro, 2016	9/10	*X*	*X*	*X*	*X*	*X*		*X*	*X*	*X*	*X*	*X*
Dreyer, 2013	6/10	*X*	*X*		*X*	*X*		*X*			*X*	*X*
Dreyer, 2018	6/10	*X*	*X*		*X*	*X*		*X*			*X*	*X*
Ikeda, 2018[d]	8/10	*X*	*X*	*X*	*X*	*X*			*X*	*X*	*X*	*X*
Ikeda, 2019	8/10	*X*	*X*	*X*	*X*	*X*			*X*	*X*	*X*	*X*
Muyskens, 2019	6/10	*X*	*X*		*X*	*X*		*X*			*X*	*X*
Summary[e]		6	6	3	6	6	0	4	3	3	6	6

[a]PEDro, Physiotherapy Evidence Database. Guidelines of PEDro scale are available from PEDro database (https://www.pedro.org.au/english/downloads/pedro-scale/).

[b]Points of methodological quality are denoted as "*X*" for fulfilled criteria.

[c]This item is not used to calculate the total score.

[d]Score was determined by a third assessor.

[e]This was calculated as the number of studies satisfied.

PEDro classification scale: 1 = random allocation, 2 = concealed allocation, 3 = similarity at the baseline, 4 = subject blinding, 5 = therapist blinding, 6 = assessor blinding, 7 = more than 85% follow-up for at least one key outcome, 8 = intention-to-treat analysis, 9 = between-group statistical comparison for at least one key outcome, 10 = point and variability measures for at least one key outcome. Methodological quality: high, ≥7 points; medium, 4–6 points; low, ≤3 points.

Supplementary Figure S1

Study or Subgroup	PS plus ET Mean	PS plus ET SD	PS plus ET Total	ET Mean	ET SD	ET Total	Weight	Mean Difference IV, Fixed, 95% CI
**a) Quadriceps volume (%); the operative leg; follow up >4 wk**								
Dreyer 2013, involved leg	-6.2	8.8	16	-18.4	7.97	12	33.9%	12.20 [5.96, 18.44]
Dreyer 2018, involved leg	-8.5	6.1	19	-13.4	8.05	20	66.1%	4.90 [0.43, 9.37]
**Subtotal (95% CI)**			**35**			**32**	**100.0%**	**7.38 [3.74, 11.01]**
Heterogeneity: Chi² = 3.48, df = 1 (P = 0.06); I² = 71%								
Test for overall effect: Z = 3.98 (P < 0.0001)								
**b) Quadriceps volume (%); the contralateral leg; follow up >4 wk**								
Dreyer 2013, uninvolved leg	-2.9	7.2	16	-9.6	6.24	12	41.1%	6.70 [1.71, 11.69]
Dreyer 2018, uninvolved leg	-1.5	6.97	19	-7.2	6.26	20	58.9%	5.70 [1.53, 9.87]
**Subtotal (95% CI)**			**35**			**32**	**100.0%**	**6.11 [2.91, 9.31]**
Heterogeneity: Chi² = 0.09, df = 1 (P = 0.76); I² = 0%								
Test for overall effect: Z = 3.75 (P = 0.0002)								

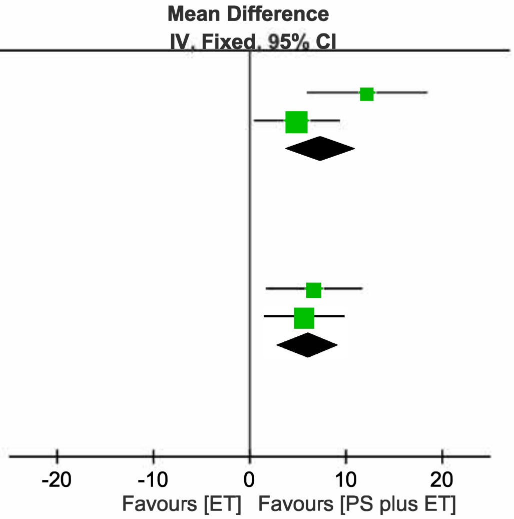

Figure S1. Forest plot summarizing the effects of postoperative protein supplementation (PS) plus exercise training (ET) on changes in quadriceps muscle volume of (**a**) the involved leg and (**b**) the contralateral uninvolved leg over medium-term follow-up. For each trial, the square represents the point estimate of the intervention effect. The horizontal line links the lower and upper limits of the 95% CI of this effect. The area of the squares reflects the relative weight of the trials in the meta-analysis. Trial results plotted on the right-hand side of the vertical axis indicate effects in favor of protein supplementation. The combined effects are plotted using black diamonds.
95% CI, 95% confidence interval; Std, standard; IV, inverse variance.

Supplementary Figure S2

Study or Subgroup	PS plus ET Mean	PS plus ET SD	PS plus ET Total	ET Mean	ET SD	ET Total	Weight	Mean Difference IV, Fixed, 95% CI
**a) Hamstrings volume (%); operative leg; follow up >4 wk**								
Dreyer 2013, involved leg	-7.5	6.8	16	-18.2	6.24	12	49.3%	10.70 [5.85, 15.55]
Dreyer 2018, involved leg	-7.4	8.72	19	-12.2	6.26	20	50.7%	4.80 [0.01, 9.59]
**Subtotal (95% CI)**			**35**			**32**	**100.0%**	**7.71 [4.30, 11.12]**
Heterogeneity: Chi² = 2.88, df = 1 (P = 0.09); I² = 65%								
Test for overall effect: Z = 4.43 (P < 0.00001)								
**b) Hamstrings volume (%); contralateral leg; follow up >4 wk**								
Dreyer 2013, uninvolved leg	-1.6	5.2	16	-8.3	4.85	12	51.9%	6.70 [2.96, 10.44]
Dreyer 2018, uninvolved leg	-2.1	5.67	19	-7.5	6.71	20	48.1%	5.40 [1.51, 9.29]
**Subtotal (95% CI)**			**35**			**32**	**100.0%**	**6.08 [3.38, 8.77]**
Heterogeneity: Chi² = 0.22, df = 1 (P = 0.64); I² = 0%								
Test for overall effect: Z = 4.41 (P < 0.0001)								

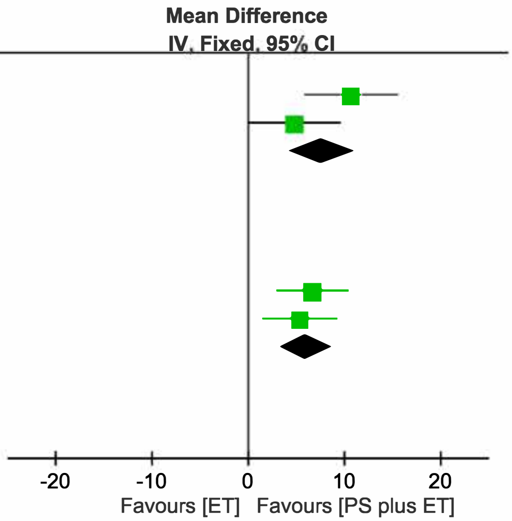

Figure S2. Forest plot summarizing the effects of postoperative protein supplementation (PS) plus exercise training (ET) on changes in hamstring muscle volume of (**a**) the involved leg and (**b**) the contralateral uninvolved leg over medium-term follow-up. For each trial, the square represents the point estimate of the intervention effect. The horizontal line links the lower and upper limits of the 95% CI of this effect. The area of the squares reflects the relative weight of the trials in the meta-analysis. Trial results plotted on the right-hand side of the vertical axis indicate effects in favor of protein supplementation. The combined effects are plotted using black diamonds.
95% CI, 95%confidence interval; Std, standard; IV, inverse variance.

Supplementary Figure S3

Study or Subgroup	PS plus ET Mean	PS plus ET SD	PS plus ET Total	ET Mean	ET SD	ET Total	Mean Difference
**a) Upper arm CSA (cm^2)**							
Ikeda 2019	-1.4	1.2	18	-2.8	1.8	13	1.40 [0.28, 2.52]
**b) Number of myofiber of quadriceps**							
Muyskens 2019, uninvolved leg	5.16	10.74	7	-25	11.34	9	19.84 [8.97, 30.71]
Muyskens 2019, involved leg	15.05	37.52	8	9.1	15.61	5	5.95 [-23.43, 35.33]

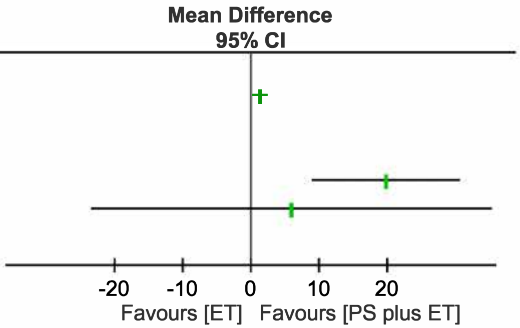

FigureS3. Forest plot summarizing the effects of postoperative protein supplementation (PS) plus exercise training (ET) on changes in (**a**) upper-arm CSA and (**b**) number of quadriceps myofibers over the overall follow-up period. For each trial, the square represents the point estimate of the intervention effect. The horizontal line links the lower and upper limits of the 95% CI of this effect. Trial results plotted on the right-hand side of the vertical axis indicate effects in favor of protein supplementation.
95% CI, 95% confidence interval; Std, standard; IV, inverse variance; CSA, cross-sectional area.

Supplementary Figure S4

Study or Subgroup	PS plus ET Mean	PS plus ET SD	PS plus ET Total	ET Mean	ET SD	ET Total	Mean Difference
**Hip abductor muscle strength (%), non-TJA**							
Ikeda 2018, involved leg	8.9	21.6	21	-0.3	14.2	22	9.20 [-1.78, 20.18]
Ikeda 2018, uninvolved leg	14.2	19.4	21	-2.6	16.5	22	16.80 [6.01, 27.59]

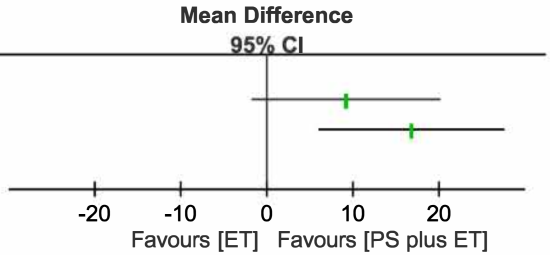

Figure S4. Forest plot summarizing the effects of protein supplementation (PS) plus exercise training (ET) on changes in hip abductor strength in patients who did not undergo total joint arthroplasty. For each trial, the square represents the point estimate of the intervention effect. The horizontal line links the lower and upper limits of the 95% CI of this effect. Trial results plotted on the right-hand side of the vertical axis indicate effects in favor of protein supplementation.
95% CI, 95% confidence interval; Std, standard; IV, inverse variance; TJA, total joint arthroplasty.

Supplementary Figure S5

Study or Subgroup	PS plus ET Mean	PS plus ET SD	PS plus ET Total	ET Mean	ET SD	ET Total	Mean Difference	Mean Difference 95% CI
**Timed 10-m walk (%), non-TJA**								
Ikeda 2018	10	13.6	21	6.6	10.1	22	3.40 [-3.79, 10.59]	

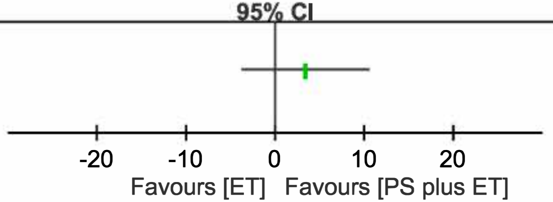

Figure S5. Forest plot summarizing the effects of protein supplementation (PS) plus exercise training (ET) on changes in walking speed in patients who did not undergo total joint arthroplasty. For each trial, the square represents the point estimate of the intervention effect. The horizontal line links the lower and upper limits of the 95% CI of this effect. Trial results plotted on the right-hand side of the vertical axis indicate effects in favor of protein supplementation.
95% CI, 95% confidence interval; Std, standard; IV, inverse variance; TJA, total joint arthroplasty.

Supplementary Figure S6

Study or Subgroup	PS plus ET Mean	PS plus ET SD	PS plus ET Total	ET Mean	ET SD	ET Total	Weight	Mean Difference IV, Fixed, 95% CI
**C-Reactive protein (mg/L), overall follow up duration**								
Baldissarro 2016	0.92	0.65	30	0.78	0.4	30	31.4%	0.14 [-0.13, 0.41]
Dreyer 2018	0.08	0.13	19	-0.09	0.4	20	68.6%	0.17 [-0.01, 0.35]
**Subtotal (95% CI)**			**49**			**50**	**100.0%**	**0.16 [0.01, 0.31]**

Heterogeneity: Chi² = 0.03, df = 1 (P = 0.86); I² = 0%
Test for overall effect: Z = 2.06 (P = 0.04)

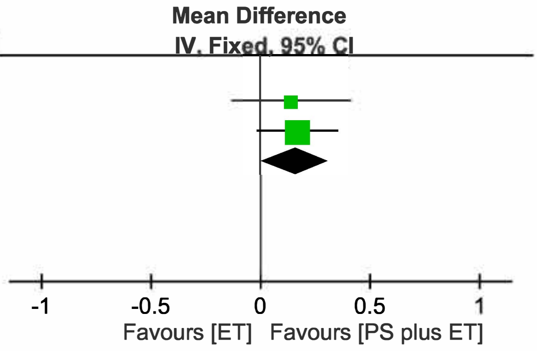

Figure S6. Forest plot summarizing the effects of postoperative protein supplementation (PS) plus exercise training (ET) on changes in C-reactive protein over the overall follow-up period. For each trial, the square represents the point estimate of the intervention effect. The horizontal line links the lower and upper limits of the 95% CI of this effect. The area of the squares reflects the relative weight of the trials in the meta- analysis. Trial results plotted on the right-hand side of the vertical axis indicate effects in favor of protein supplementation. The combined effects are plotted using black diamonds.
95% CI, 95% confidence interval; Std, standard; IV, inverse variance.

Supplementary Figure S7

Study or Subgroup	PS plus ET Mean	PS plus ET SD	PS plus ET Total	ET Mean	ET SD	ET Total	Mean Difference
**a) Interleukin-6, follow up≤4 wk**							
Muyskens 2019	0.6	8.05	19	-1.98	6.21	22	2.58 [-1.87, 7.03]
**b) TNF-α, follow up≤4 wk**							
Muyskens 2019	-1.35	5.12	19	-2.95	4.28	22	1.60 [-1.32, 4.52]

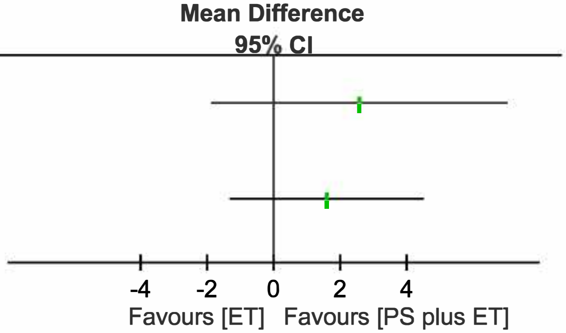

Figure S7. Forest plot summarizing the effects of postoperative protein supplementation (PS) plus exercise training (ET) on changes in (**a**) interleukin-6 and (**b**) TNF-α over short-term follow-up. For each trial, the square represents the point estimate of the intervention effect. The horizontal line links the lower and upper limits of the 95% CI of this effect. Trial results plotted on the right-hand side of the vertical axis indicate effects in favor of protein supplementation.
95% CI, 95% confidence interval; Std, standard; IV, inverse variance; TNF-α, tumor necrosis factor-α.

Supplementary Figure S8

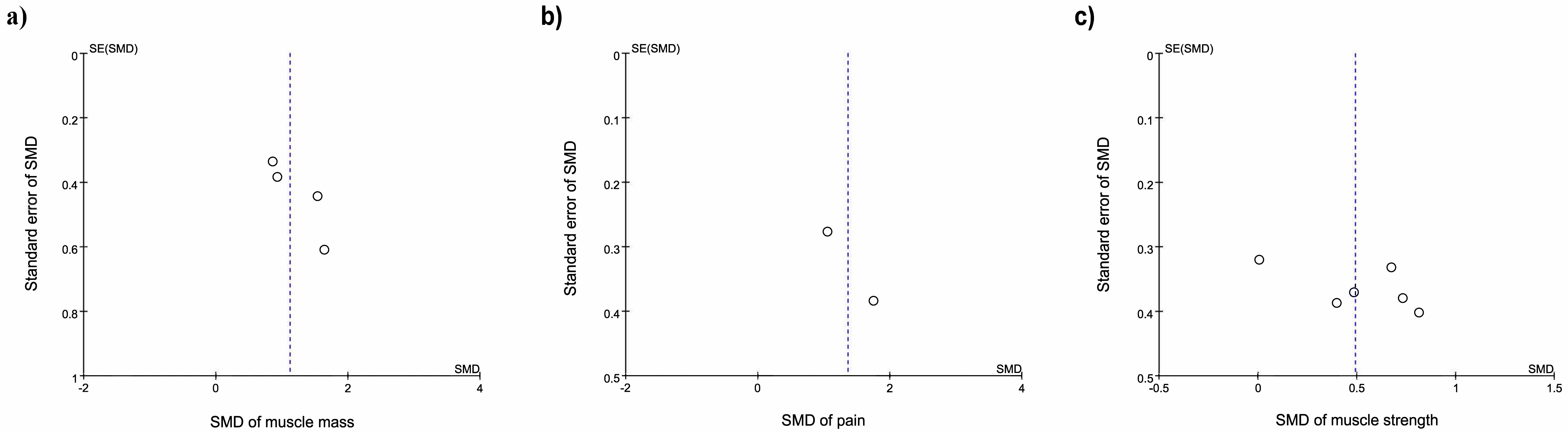

Figure S8. Funnel plots of intervention effects for (**a**) muscle mass, (**b**) pain, and (**c**) muscle strength. Each circle denotes an independent comparison, with the X-axis representing a standard mean difference (SMD) over control comparisons and the Y-axis depicting the standard error (SE) of SMD. The vertical dotted line indicates the SMD of the combined effect for each outcome measure.
